# Supplementary material for: Isolation, Characterization and Antibacterial Activity of 4-Allylbenzene-1,2-diol from Piper austrosinense
Source: Molecules. 2023 Apr 19;28(8):3572. doi: 10.3390/molecules28083572 (PMC10146670; doi:10.3390/molecules28083572)
Supplement: Supplementary file 1 [file molecules-28-03572-s001.zip › molecules-2317274-supplementary.pdf]

Supplementary Materials

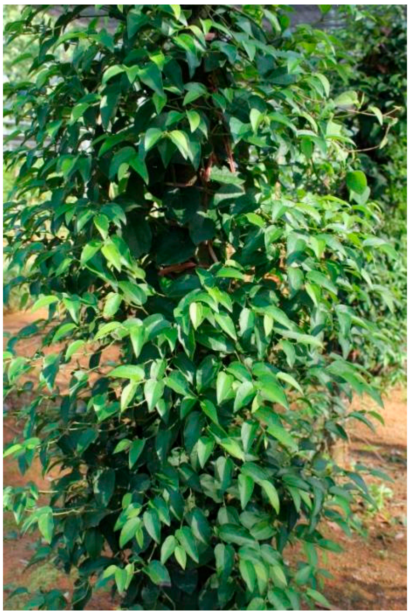

Figure S1. Picture of *Piper austrosinense.*

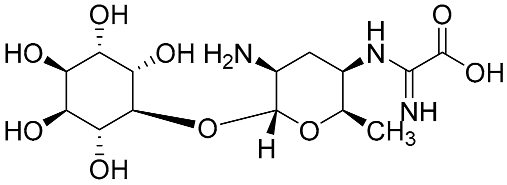

Figure S2. The chemical structures of kasugamycin.
